# Supplementary material for: A methodology for the design of experiments in computational intelligence with multiple regression models
Source: PeerJ. 2016 Dec 1;4:e2721. doi: 10.7717/peerj.2721 (PMC5136129; doi:10.7717/peerj.2721)
Supplement: Data S1 — Datailed results from UC Irvine Machine Learning Repository (Housing, Machine CPU, Wine Quality, Automobile and Parkinson) and the 3 Use Cases (Protein Corona, Gajewicz Metal Oxides and Aquatic Toxicity) [file peerj-04-2721-s001.zip › 6_UseCases/2_GajewiczMetalOxides/SVMRFE.details.csv.repeatedcv.split1.pdf]

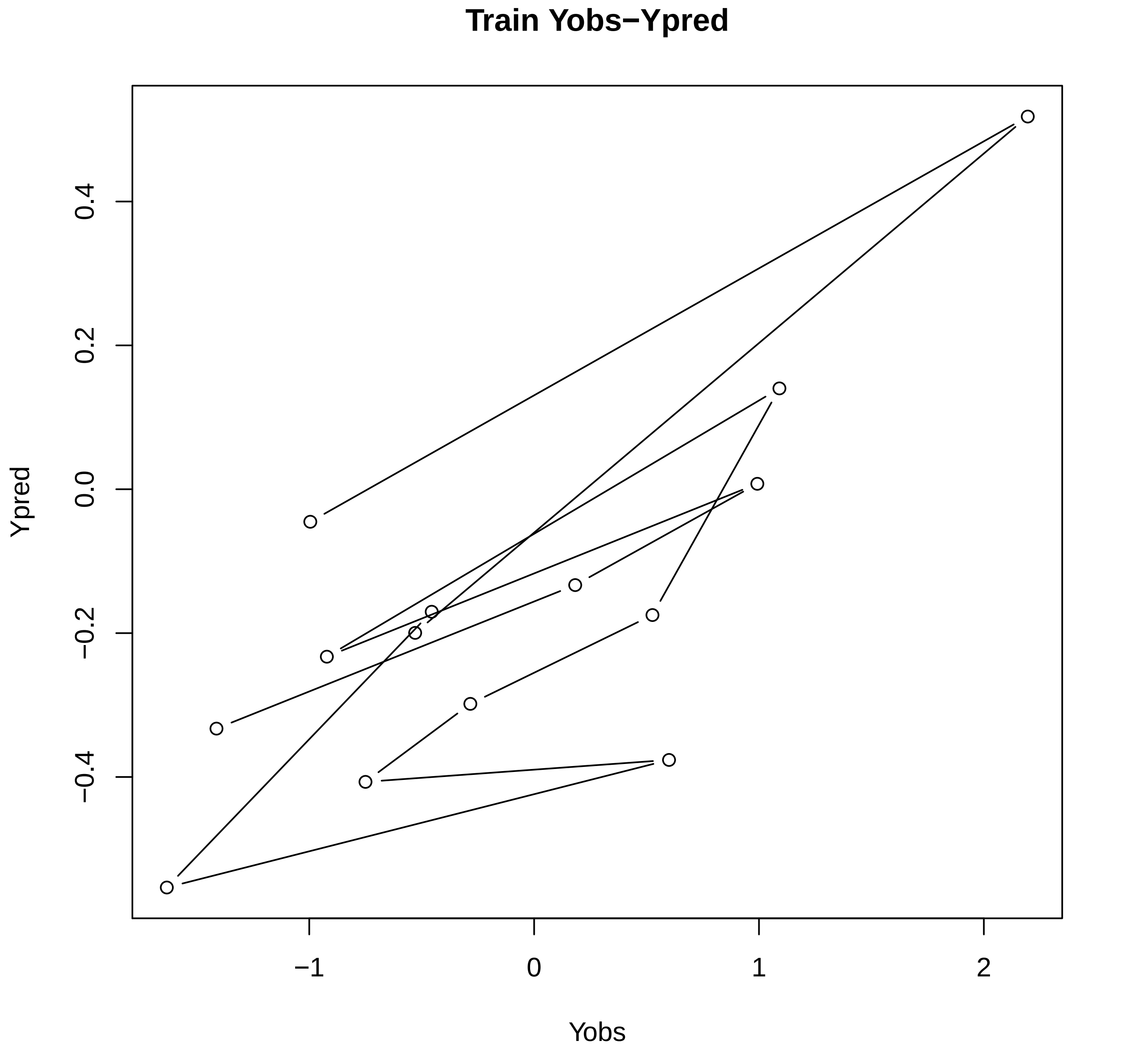

Train Yobs−Ypred
0.4
0.2
0.0
−0.2
−0.4
Ypred
−1
0
1
2
Yobs

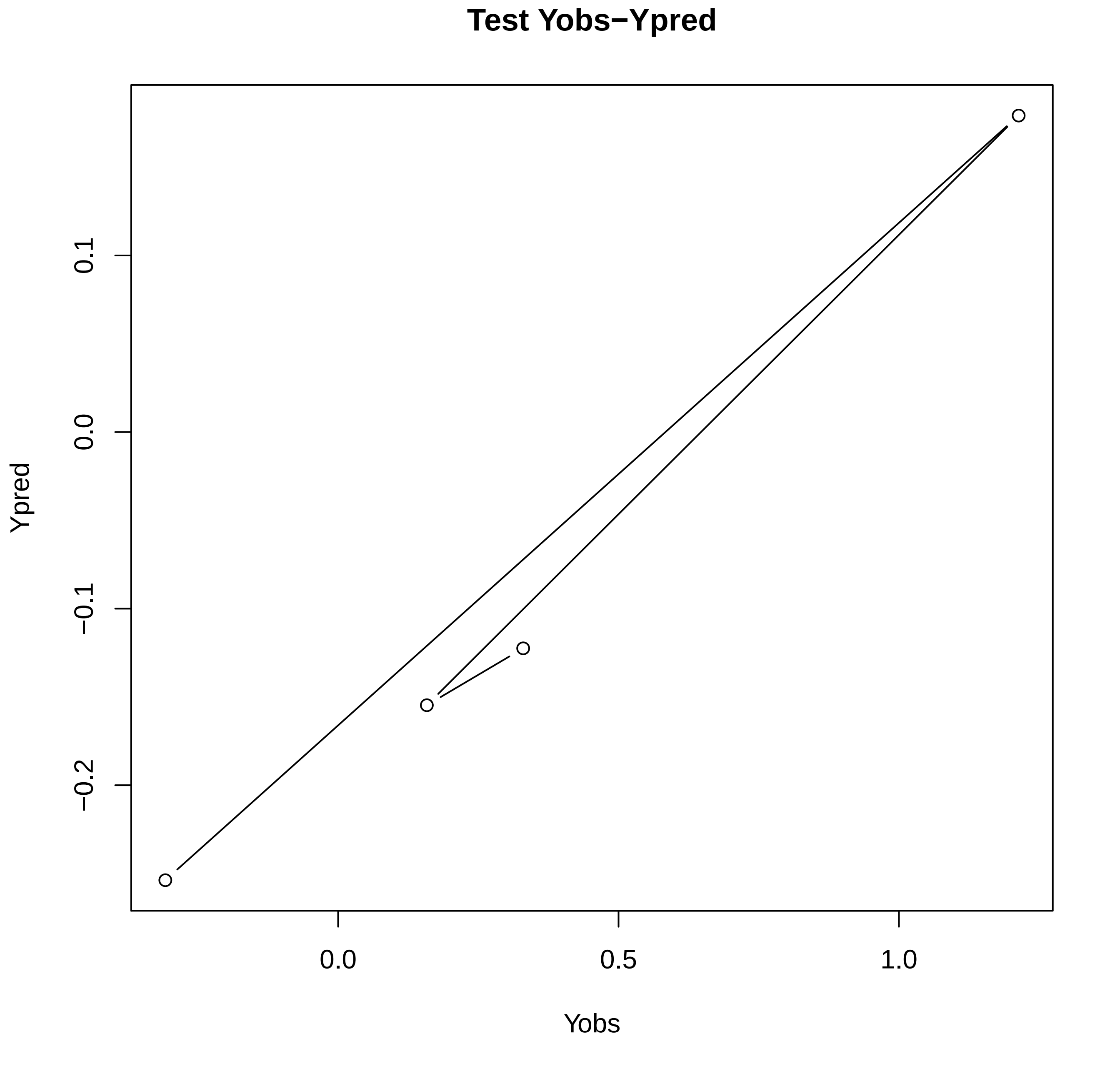

Test Yobs−Ypred
Ypred
0.1
0.0
−0.1
−0.2
0.0
0.5
1.0
Yobs

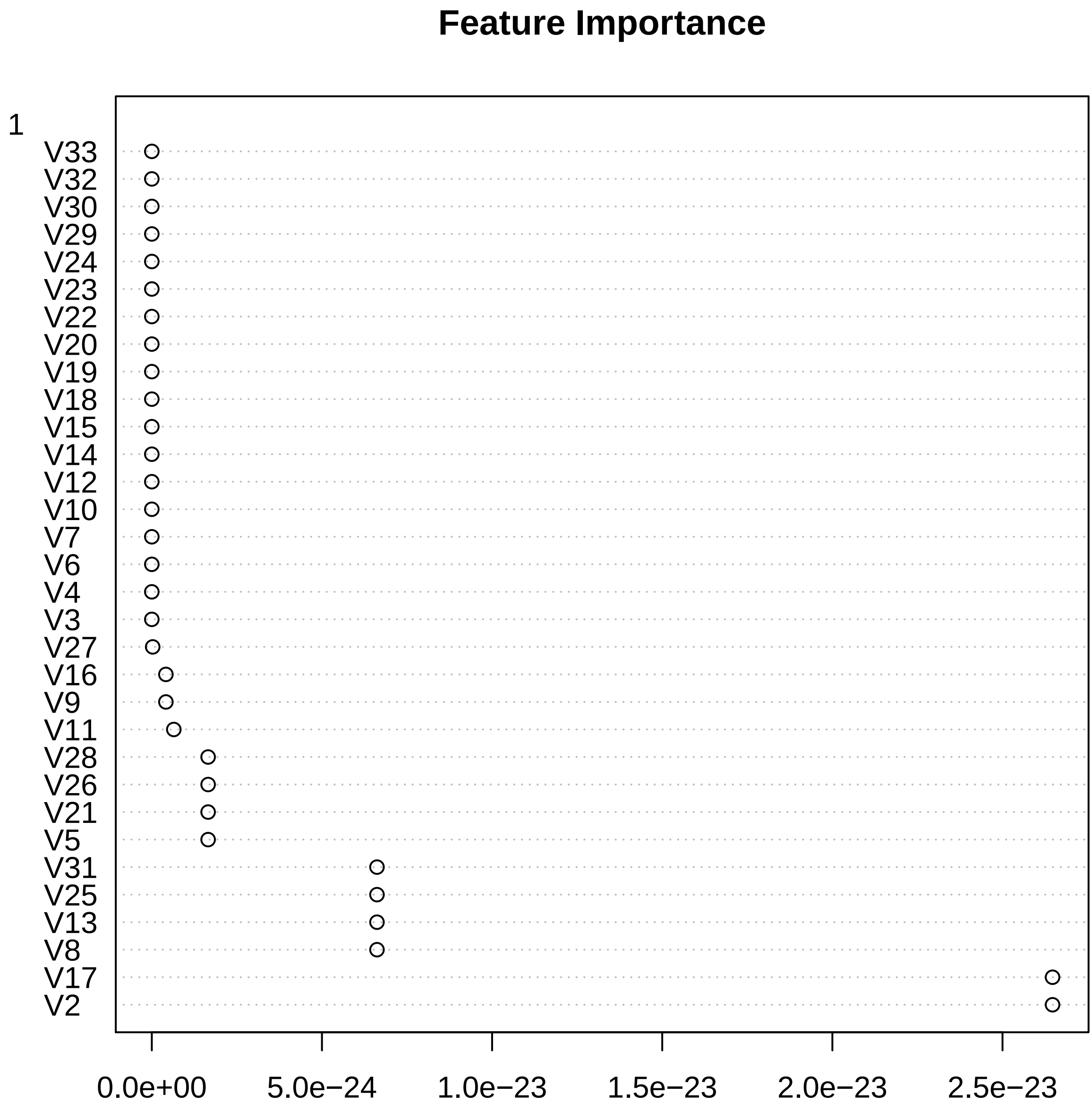

Feature Importance
1
V33
V32
V30
V29
V24
V23
V22
V20
V19
V18
V15
V14
V12
V10
V7
V6
V4
V3
V27
V16
V9
V11
V28
V26
V21
V5
V31
V25
V13
V8
V17
V2
0.0e+00
5.0e−24
1.0e−23
1.5e−23
2.0e−23
2.5e−23

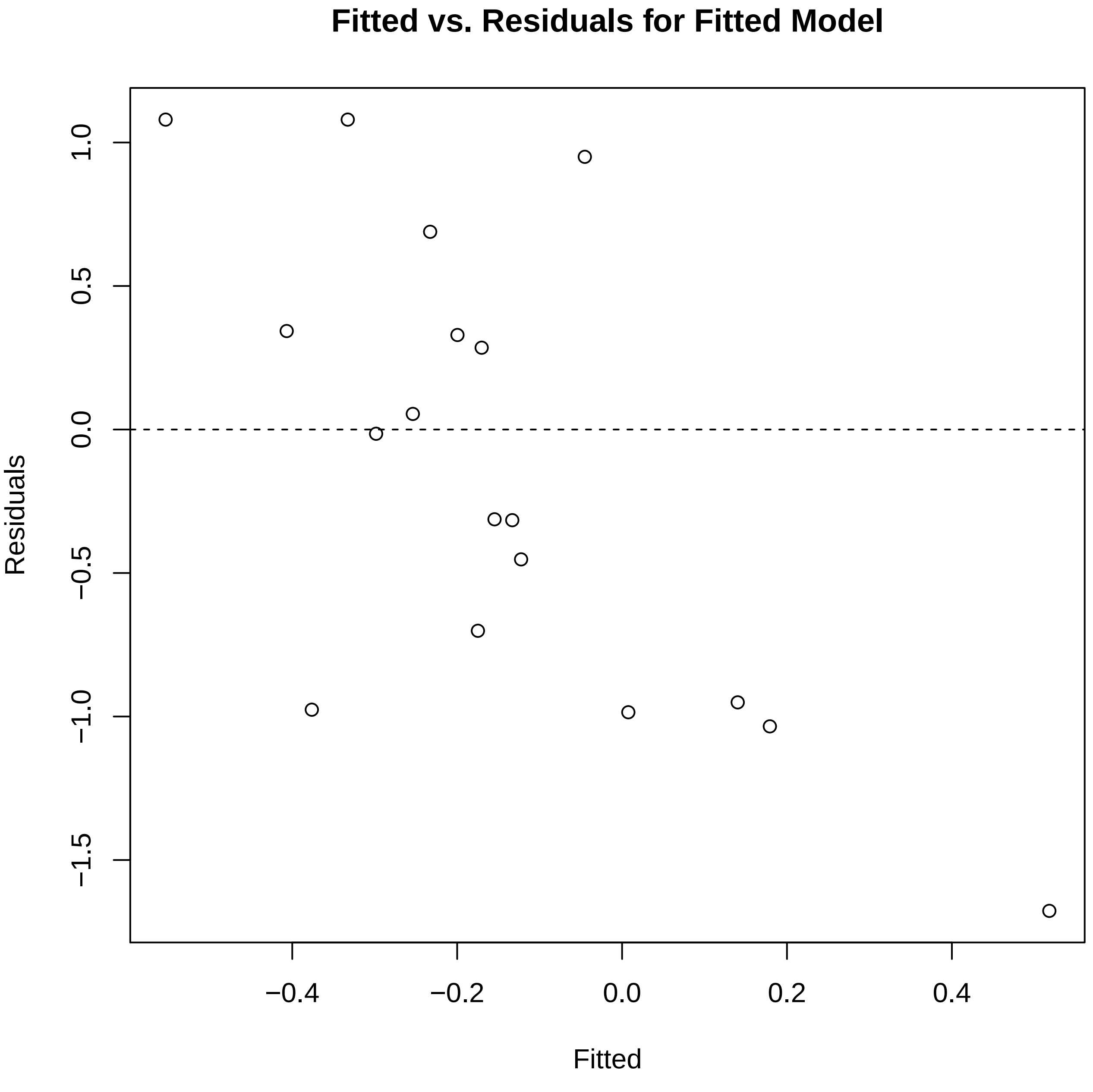
Fitted vs. Residuals for Fitted Model
Residuals
1.0
0.5
0.0
−0.5
−1.0
−1.5
−0.4
−0.2
0.0
0.2
0.4
Fitted
